# High-speed and high-contrast two-channel all-optical modulator based on solution-processed CdSe/ZnS quantum dots

**DOI:** 10.1038/s41598-022-17084-4

**Published:** 2022-07-27

**Authors:** Hannaneh Dortaj, Mohammad Faraji, Samiye Matloub

**Affiliations:** Quantum Photonics Research Lab (QPRL), University of Tabriz, Tabriz, 5166614761 Iran

**Keywords:** Quantum dots, Optoelectronic devices and components

## Abstract

Recently, all-optical modulators are potentially the most promising candidate to achieve high-bit rate modulation in high-speed all-optical communication technologies and signal processing. In this study, a two-channel all-optical modulator based on a solution-processed quantum dot structure is introduced for two sizes of quantum dots to operate at two wavelengths of MIR spectra (3 µm and 5 µm). To perform numerical and theoretical analysis and evaluate the optical behavior of the proposed all-optical modulator, the coupled rate and propagation equations have been solved by considering homogeneous and inhomogeneous broadening effects. The modulation depth at the 50 GHz frequency and 3 mW probe power is attained, about 94% for channel-1 with the wavelength of 559 nm at 300 Wcm^−2^ pump power density as well as approximately 83.5% for channel-2 with the wavelength of 619 nm at 500 Wcm^−2^ pump power density. The introduced two-channel all-optical modulator can operate simultaneously at two wavelengths during the modulation process in which information could be transmitted through both signals from the control light. This approach can present the practical device as a high-contrast and high-speed two-channel all-optical modulator with a high modulation depth in numerous applications such as thermal imaging in night vision cameras, wavelength de-multiplexing, signal processing, free-space communication.

## Introduction

The ever-increasing demand to transmit a vast amount of data has been felt since 1993 that was when the internet became available to the public around the world. The fiber-optic network with a natural transmission medium enjoys a large bandwidth, so that standard single-mode fibers have a bandwidth of up to 25 THz, requiring a very high bit rate. Nowadays, fiber optic networks worldwide operate at a bit rate of 40 Gbps, and state-of-the-art technology is always moving towards enhancing bit rates and high speeds^1,2^. Hence, high-speed optical communication technologies and signal processing are chiefly reliant on devices mentioned as optical modulators. In fact, modulators play a substantial role in optical communication compared to other components. Utilizing these components, information is transmitted on optical beams after traveling for miles, and then it is conveyed by optical fibers to the center of the telecommunication and data-sharing networks^3,4^. Over the past few years, optical modulators have a significant role in optoelectronics and photonic devices due to their large bandwidth as well as low loss, and attained enormous development in optical information processing, optical interconnects, pulsed laser engineering, and environmental sensing^5–9^.

All-optical modulators (AOMs) are potentially the most promising candidate to achieve high-bit rate modulation, in which light is modulated by light1^10–13^ and has unique advantages in all-optical signal processing compared to electro-optic modulation or acousto-optic modulation^14,15^. Recently, all-optical modulation has been likely to enable fast photonic networks due to eliminating the conversion process between light and electricity in conventional optical switching^4,10^. The AOMs have been widely inspected for their broad bandwidth, fast response and compact size, in which the light signal can be modulated in the photonic domain without exerting any external thermal, electronic, and other effects^5,8,9^. The progress of AOM goals not only at increasing the performance speed, but also at providing novel applications, such as broadband free-space communication, anti-fluctuation atmosphere imaging, and ultrafast time-of-flight detection, based on the current MIR detection technologies^16,17^.

For active controlling of light, there have been numerous interesting applications by applying semiconductor quantum dots (QDs)^4,10,18^. Recently, studies on AOMs at the telecommunication wavelength have been thoroughly developed and we have witnessed noticeable advances in this field. To this end, an experimental demonstration of AOM based on the efficient interaction between the control light at a wavelength 515 nm and the signal light at 1426 nm was reached by converting them into co-propagating surface plasmon polaritons (SPPs), which interact through a thin layer of CdSe QDs. The optical modulation at low power densities (∼ 100 Wcm^−2^) and modulation frequency around 25 MHz was observed due to high SPP field confinement and high QD absorption cross section^13^. For promoting the modulation depth (MD) and modulation frequency at this range of wavelengths a novel procedure has also been hypothetically reported to design an AOM based on CdSe-QDs-doped glass in which the strong (560 Wcm^−2^) pump light at wavelength of 460 nm was used to modulate the signal light at 1522 nm with MD of 96% and modulation frequency of 70 GHz^4^. Thanks to unique optical properties of 2D materials in development of light-control-light operations, a graphene-clad microfiber AOM can be experimentally achieved with a MD of 38% and modulation frequency of 200 MHz where the signal light at wavelength of 1550 nm was controlled by a light at 1064 nm^19^. Additionally, an AOM using a spatial cross phase modulation method based on MXene has been designed in which a strong control light (∼ 40 Wcm^−2^) at wavelength 671 nm was exploited to modulate another weak signal light at 532 nm^20^.

So far, the range of telecommunication wavelengths has been the focus of most achievements and the researchers’ studies in the field of silicon optical modulators. The mid-infrared (MIR) to far-infrared (FIR) wavelength region has attracted less attention, in spite of its great potential and great solicitation on the market^16,21^. The most prominent applications in MIR, including free-space communication, security countermeasures, and thermal imaging employ the high transmission of the Earth’s atmosphere in the wavelength range between 3.5 μm and 5 μm. The longer wavelength ensures less scattering compared to the near-infrared (NIR) and visible light, as well as guarantees no active vibrational transitions of H_2_O and CO_2_ molecules, allowing it to be remotely sensed and detected with negligible impact from climatic conditions^16,22^. In this direction, a 50-MHz AOM was demonstrated consisting of germanium on silicon waveguides at the MIR wavelength range (2 ∼ 3 μm), with MD of 60%^23^. Besides, through using an optical membrane made of silicon, an AOM theoretically has been presented within which the MD is promoted to 80% using the pump fluence of 3.8 mJ/cm^2^ and operating in the MIR ranging between 4 μm and 6 μm^16^. Newly, a niobium carbide (Nb_2_C) two-channel AOM assisted by Nb_2_CPVA film based on the thermo-optic effect was experimentally designed. The research outcomes depict that the Nb_2_C AOM has successfully transmitted the optical data from the control light at wavelengths 980 nm and 793 nm to the signal light at two telecommunication wavelengths 1.5 μm and 2.0 μm, respectively. The MD of this system is 23.3% and the highest modulation frequency is 5 KHz which provides low speed as well as a low MD^24^.

Solution-processed materials like colloidal quantum dots (CQDs) proffer higher absorption, room temperature processing, low-cost manufacturing, and ease of large-area fabrication on rigid or flexible substrates^25–28^. Having the solution-processed CQD to be considered, a novel design for high-speed and high contrast two-channel AOMs has been proposed in this paper to achieve controlling two weak signal lights at MIR spectrum range by two strong control visible light simultaneously. In the quantum-based systems, the optical absorption spectra of the QDs can be widely mastered by the quantum size effect to attain tunable absorption^25,29^. Hence, in the introduced two-channel AOM, two sizes of QDs are adjusted to absorb the two wavelengths of visible light as pump signals, to modulate two wavelengths of MIR spectrum as probe signals, respectively. One of the most promising aspects of this two-channel modulator is the possibility of simultaneously executing at two wavelengths during modulation process.

Last but not least, the two-channel AOM can operate at the high frequencies up to 50 GHz, providing a high MD > 80% and performing far better than previously reported studies. Therefore, this modulator can be utilized in numerous applications, including thermal imaging in night vision cameras, wavelength demultiplexing (WDM), signal processing, etc. Besides, the proposed AOM could be developed for more sizes of QDs to achieve multi-channel modulation.

## The proposed two-channel AOM

**Figure 1:**
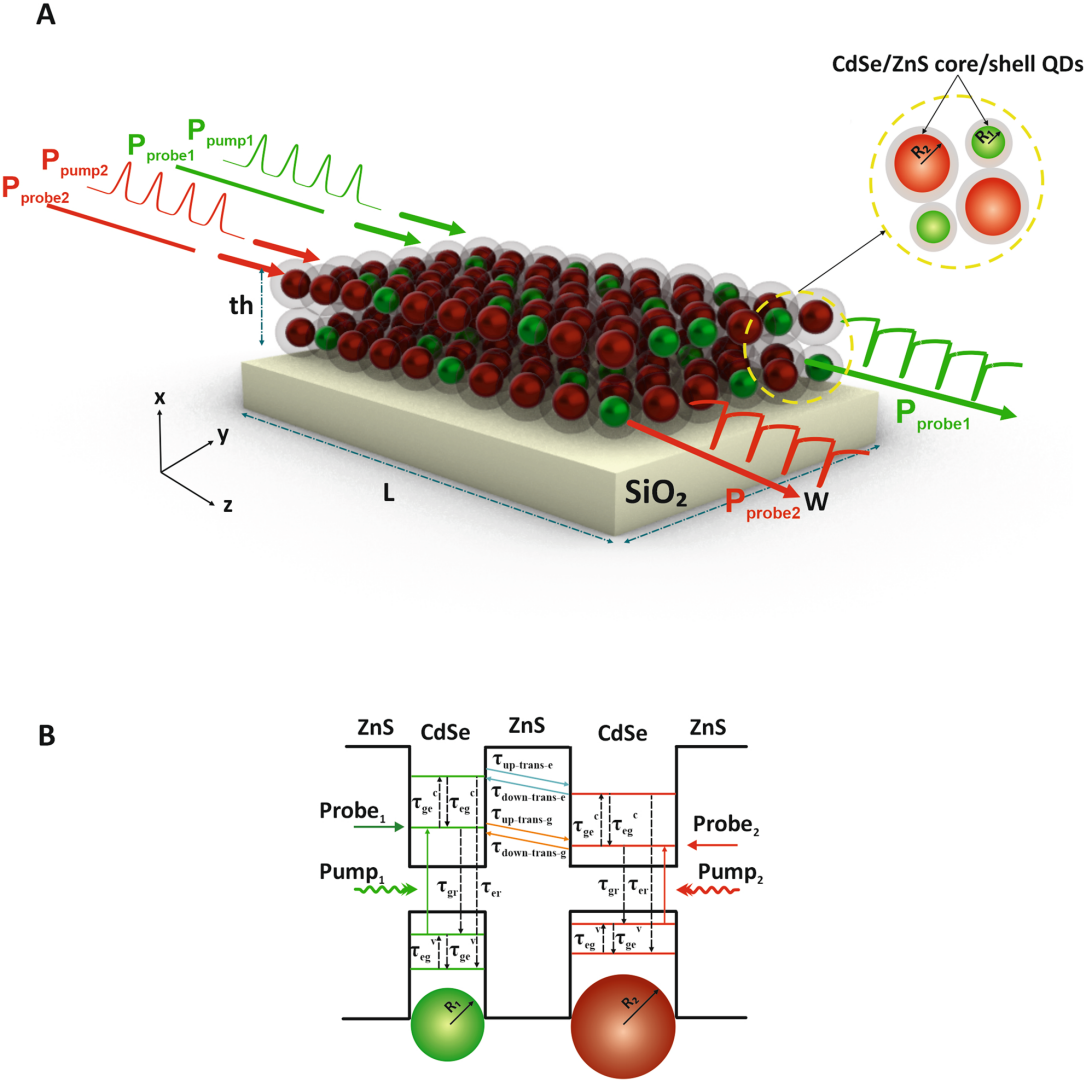
The schematic of the proposed two-channel AOM. (**A**) Schematic of an array of two size of QDs core/shell CdSe/ZnS structure. (**B**) The energy band diagram of the proposed structure including the carrier absorption, recombination process, and the fluorescence resonance energy transfer (FRET) into two sizes of QDs.

In this research, a high-speed and high-contrast two-channel AOM utilizing two different groups of QDs synthesized by the solution process technology has been proposed. The schematic of the proposed two-channel AOM is depicted in Fig. 1A, in which pump and probe signals are applied through two different channels. Among all criteria to design a modulator, selecting nanomaterial as an absorber is significant.

Hence, to realize two-channel modulation, a core/shell structure including an array of two different sizes of CdSe QDs surrounded by a ZnS shell as the visible light absorber is exploited with radiuses of R_1_ and R_2_ arranged randomly and irregularly and corresponding to channel-1 (green QDs) and channel-2 (red QDs), respectively.

Carrier transitions between ground states of the conduction band and the valence band play a key role in the modulation process^4^. Two laser beams, including the modulated pump signals with the wavelengths of 559 nm (green) and 619 nm (red), are applied as information signals, as well as two continuous waves (CW) laser beams, including the probe signals with the wavelengths of 3 μm and 5 μm (MIR spectrum) are simultaneously applied which are set for the QD radiuses of R_1_ = 2 nm and R_2_ = 2.8 nm, respectively.

The absorption and recombination process by considering relaxation time constants has been illustrated in Fig. 1B. As indicated in this figure, the energy band diagram includes the ground state of the conduction band (GS^c^), the excited state of the conduction band (ES^c^), and the ground state of the valence band (GS^v^), and the excited state of the valence band (ES^v^) corresponds to the small green QDs (channel-1) and the big red QDs (channel-2) along with the carriers and photons dynamics that describe the performance of the two-channel AOM. Each probe signal is absorbed as a result of the intersubband transition from GS^c^ to ES^c^ inside the related QD groups, provided that the carriers had been excited by the pump signals due to interband absorption between GS^v^ and GS^c^ inside corresponding groups of QDs. Besides, the time constants related to the relaxation process of carriers in each QD group are indicated in Fig. 1B as *τ*_eg_^c^ (electron relaxation time from the ES^c^ to the GS^c^), *τ*_ge_^v^ (electron relaxation time from the GS^v^ to the ES^v^), *τ*_gr_ (electron recombination time from the GS^c^ to the GS^v^), *τ*_er_ (electron recombination time from the ES^c^ to the ES^v^), the time constants related to FRET mechansim are demonstrated as *τ*_down-trans-g(e)_ (electron transition time from the GS^c^ (ES^c^) of channel-1 to the GS^c^ (ES^c^) of channel-2) and *τ*_up-trans-g(e)_ (electron transition time from the GS^c^ (ES^c^) of channel-2 to the GS^c^ (ES^c^) of channel-1).

In the QD-based systems, carrier recombination lifetime is around nanoseconds, which is an obstacle to reaching high-speed devices. It is verified that different from the electron relaxation time that depends on the solvent in which it is grown, the hole relaxation time is independent of nanocrystal surface treatment^30^. Furthermore, due to increasing the surface-to-volume ratio by reducing the QD size, the relaxation rates ascend^31,32^ which leads to fast carrier trapping in surface states. Even though the space between energy levels becomes larger by decreasing the QD size, the electron recombination lifetime is descend^32^. Therefore, the electron recombination time of trap states is considered in the calculation of τ_gr_ and τ_er_ in which these values are decreased from nanosecond to picosecond to obtain high-frequency two-channel AOM^4,30,33^.

Regarding the environmental parameters of the system, the effective area of the incident light source is 200 μm × 200 μm, and the temperature is 300 K. The laser beam is typically incident on the side surface of the device where it passes the length of L. The light that touches the modulator surface is monochromatic.

## The theoretical modeling of two-channel AOM

This section contains four subsections. First, for modal analyzing of two-channel AOM the 3D Schrodinger equation has solved to attain eigen-energies and wavefunctions. Therefore, the energy band diagram of simulated structure can be achieved to model the absorption and recombination process. Second, the homogeneous and inhomogeneous broadening arising from intrinsic effects such as pressure or temperature and non-uniformity of QD sizes as a result of solution-process method are applied on intersubband and interband absorption coefficients. Third, the fluorescence resonance energy transfer (FERT) due to using of two different sizes of quantum dots in the two-channel AOM, occurs between two QDs. Therefore, these transitions are included in the coupled rate equations. Forth, for analyzing the performance of two-channel AOM and characterizing the modulator’s essential factors the coupled rate and propagation equations are solved by finite difference time domain (FDTD) method.

### The modal analysis of the two-channel AOM

Depending on the effective mass approximation, eigen-energies and the corresponding wavefunctions of the GS^c^, ES^c^, GS^v^, and ES^v^ have been obtained by solving 3D Schrodinger equation exploiting the software based on the Finite Element Method using the parameters referred in Table 1. For expanding of the simulated structure to the practical designed device, the periodic boundary condition is applied.

**Table 1:** Material parameters required in solving Schrodinger Equation^53–57^.

Discription	Symbol	Materials	
		CdSe	ZnS
Electron affinity	*χ* [eV]	4.95	3.8
Bandgap energy	E_g_ [eV]	1.68	3.72
Effective mass of electron	*m*_e_*	0.13*m*_0_	0.22*m*_0_
Effective mass of hole	*m*_h_*	0.3*m*_0_	1.76*m*_0_

**Figure 2:**
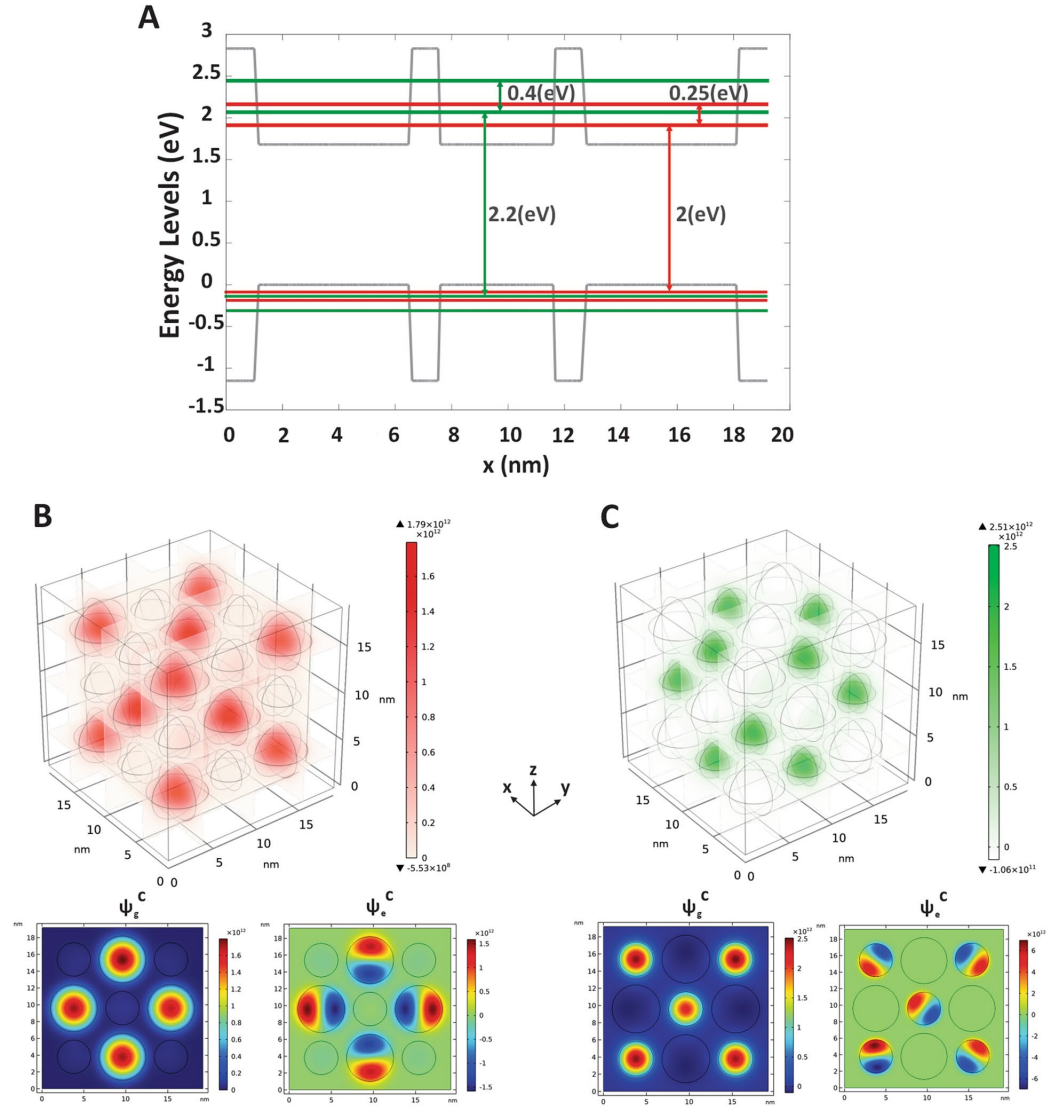
The modal analysis of two-channel AOM. (**A**) Band structure of the CdSe/ZnS core/shell two-channel AOM which indicates that the difference between the ground states of the valence and conductive bands in defined QDs is about 2.2 eV for channel 1 and 2 eV for channel 2. Also, the difference between the ground state and excited state of the conductive bands is 0.4 eV for channel 1 and 0.25 eV for channel 2. (**B**) Wave functions of CdSe QDs simulated by solving 3D Schrodinger equation in FEM software. A cross section of 3D core/shell structure in x–y plane is specified and the wave function of GS^c^ s (left) and ES^c^ s (right) of main mode is illustrated for channel-1 and (**C**) for channel-2.

The energy band diagram of the system has been illustrated in Fig. 2A. The green and red line each corresponds to the eigen-energy of green and red QD groups, respectively. As Fig. 2A shows, the difference between the GS^v^ and GS^c^ in defined QDs is about 2.2 eV for channel-1 with the radius of R_1_ and 2 eV for channel-2 with the radius of R_2_, suitably tuned for interband absorption of light beam at the green and red wavelengths, respectively. Also, the difference between the GS^c^ and ES^c^ is 0.4 eV for channel-1 and 0.25 eV for channel-2, which is suitable for intersubband absorption of 3 μm and 5 μm wavelengths, respectively. The 3D calculated wavefunctions according to GS^c^ of each QD group have been shown in Fig. 2B,C. Moreover, the cross-section of the 3D plot in the x-y plane is specified, and the wave function of GS^c^ and ES^c^ of the main mode is illustrated for channel-1 and channel-2 in Fig. 2B,C.

### The homogeneous and inhomogeneous broadening

For a modulator, the evaluation criteria include MD, modulation frequency, fabrication cost, switching energy, device size, manufacturing difficulty, device compatibility with electronic or photonic technology, etc^4^. In this section, the impact of homogeneous broadening (HB) and inhomogeneous broadening (IHB) on MD of proposed structure are explained in detail. In addition, the MD as a function of pump power, probe power, and pump frequency is calculated through rate equations, and the results are illustrated in the simulation result section.

Due to the solution process method, which proffers low-cost manufacturing, higher absorption, and a simple chemical system with controllable experimental conditions, attaining the accurate size of QDs is confined because of some negligible restriction of synthesis conditions^34–37^. Therefore, the size of each QD group related to the desired wavelengths can digress from the central value of the radius resulting in the distribution of energy levels called IHB. Considering these effects of QDs size non-uniformity on the absorption spectra of the MD, the IHB of energy levels is modeled by the Gaussian function in which the QDs ensemble is divided into 2*M* + 1 groups of identical dots with an energy interval of Δ*E*=1 meV^38–41^.

1 $$E_{n,i}^{p} = E_{0,i}^{p} - (M + 1 - n)\Delta E\;\;\;n = 1,...,2M + 1\;\;\;i = 1,2\;\;p = {\text{pump,}}\;{\text{probe}}\;$$

2 $$G_{i} (E_{n,i}^{p} ) = \frac{1}{{\sqrt {2\pi } \xi_{0} }}\exp \left[ {\frac{{ - \left( {E_{n,i}^{p} - E_{0,i}^{p} } \right)^{2} }}{{2\xi_{0}^{2} }}} \right]$$

Here, $E_{0,i}^{p}$ is the interband or intersubband transition energy of the most probable size of QDs (*M* + *1*-th QD group), *n* is the index related to the number of active modes, the index of *i* is equal to 1 for channel-1 and 2 for channel-2, and ξ_0_ is QD coverage in which the Full-wave Half Maximum (FWHM) is equal to^34,38^*.* On the other hand, intrinsic effects such as pressure or temperature on all QDs lead to the HB of energy levels which homogeneously impact all groups. One of the most prominent properties in semiconductors is the evolution of the HB of exciton luminescence on temperature since it is directly associated with the interactions between excitons (carrier-carrier), phonon-carrier scattering, and the lattice vibrational modes. It is well known that such interaction leads to a temperature-dependent linewidth. The HB is modeled by a Lorentzian function with $\Gamma_{HB}$ as its FWHM which is considered as 20 meV at room temperature^38,42^.

3 $$B_{m,n} (E_{n,i}^{p} - E_{m,i}^{p} ) = \frac{1}{\pi }\frac{{\Gamma_{HB} /2}}{{\left( {E_{n,i}^{p} - E_{m,i}^{p} } \right)^{2} + (\Gamma_{HB} /2)^{2} }}\,\,\,\,\,\,\,\,\,\,\,\,\,\,n = 1, \ldots ,2M + 1$$

Considering the broadenings are obtained, the linear interband and intersubband absorption coefficients are calculated below. The interband absorption coefficient arising from applied pump power results in the transitions of the GS^v^ to GS^c^, and it is attained by^43^,

4 $$\alpha_{m,n,i}^{i - band} = \frac{1}{{V_{QD,i} }}\frac{{10^{3} \sqrt {2\pi } }}{3}\frac{{e^{2} }}{{c\hbar \varepsilon_{0} \sqrt {\varepsilon_{QD} } }}E_{n,i}^{pump} | < \Psi_{g,i}^{v} |\widehat{e}.\widehat{r}|\Psi_{g,i}^{c} > |^{2} B_{m,n} \left( {E_{n,i}^{pump} - E_{m,i}^{pump} } \right)G_{i} \left( {E_{n,i}^{pump} } \right)$$

Similarly, the intersubband absorption coefficient arising from applied probe power leads to the transitions of the ground state in the conductive band (GS^c^) to the excited state in the conductive band (ES^c^) and it is obtained through^44^,

5 $$\alpha_{m,n,i}^{i - subband} = \frac{1}{{V_{QD,i} }}\frac{{16\pi^{2} }}{3}\frac{{e^{2} }}{c\hbar }\left( {\frac{3\varepsilon }{{2\varepsilon + \varepsilon_{QD} }}} \right)^{2} E_{n,i}^{probe} | < \Psi_{g,i}^{c} |\widehat{e}.\widehat{r}|\Psi_{e,i}^{c} > |^{2} B_{m,n} \left( {E_{n,i}^{probe} - E_{m,i}^{probe} } \right)G_{i} \left( {E_{n,i}^{probe} } \right)$$

where *e* is the elementary charge, c is the free space light speed, ε0 is the free space permittivity, ħ is the reduced Planck’s constant, $\varepsilon_{QD}$ is the dielectric constant of CdSe QDs, ε is the dielectric constant of ZnS, and $V_{QD,i}$ is the volume of related single QD. The terms $| < \Psi_{g,i}^{v} |\widehat{e}.\widehat{r}|\Psi_{g,i}^{c} > |$ and $| < \Psi_{g,i}^{c} |\widehat{e}.\widehat{r}|\Psi_{e,i}^{c} > |$ are the interband and intersubband transition dipole moments, respectively in which $\Psi_{g(e)}^{v(c)}$ is depicted in Fig. 2. The polarization of incident light is set to 45 degree in which the maximum intersubband dipole moment of the CdSe QD is obtained.

### The fluorescence resonance energy transfer (FERT)

Fluorescence (or Förster) resonance energy transfer (FRET) is a non-radiative energy transfer process from a fluorescent donor to a lower energy acceptor through interactions of donor and acceptor dipole moments^45^. This mechanism is investigated with a specific attention in many applications amongst semiconductor quantum dots (QDs). The fundamentals of FRET within a nominally homogeneous QD population as well as energy transfer between two distinct colors of QDs are discussed^46^. In the proposed two-channel AOM due to using of two different sizes of QDs, this transition process is considered as a transfer rate $\left( {W_{12,n}^{l} } \right)$obtained through:

6 $$W_{12,n}^{l} = \frac{2}{{V_{eff} }}\frac{{e^{2} }}{{\hbar n_{r}^{2} \varepsilon_{0} }}E_{12,n}^{l} | < \Psi_{l,1}^{c} |\widehat{e}.\widehat{r}|\Psi_{l,2}^{c} > |^{2} B_{m,n} \left( {E_{12,m}^{l} - E_{12,n}^{l} } \right)G\left( {E_{12,n}^{l} } \right)\quad \quad l = GS^{c} ,ES^{c}$$

where, $V_{eff}$ is the effective volume of two sizes of QDs, $n_{r}$ is the refractive index of QDs and $E_{12}^{l} = E_{1}^{l} - E_{2}^{l}$. The time constants of transitions between two sizes of QDs are calculated through $\tau_{up(down)\_trans}^{l} = 1/W_{12,n}^{l}$ and included in coupled rate equations.

### The rate and propagation equations

The performance of most optoelectronic devices has usually been analyzed by solving the coupled rate equation. To this end, the characteristics of the proposed two-channel AOM have been calculated utilizing the developed coupled rate equations, along with the propagation equation for the pump and probe beams. It is good to be mentioned that the CW probe signals and the Gaussian pulse train of pump signals are applied at z = 0 and propagated along the z-direction. The output signals are calculated at z = L. Therefore, the developed rate equation and propagation equations for the two-channel AOM are expressed as:

7 $$\begin{aligned} \frac{{dn_{{g_{n,1} }}^{c} (z,t)}}{dt} & = - \frac{{n_{{g_{n,1} }}^{c} (z,t)}}{{\tau_{down\_trans\_g} }}\left( {1 - f_{{g_{n.2} }}^{c} (z,t)} \right) + \frac{{n_{{g_{n,2} }}^{c} (z,t)}}{{\tau_{up\_trans\_g} }}\left( {1 - f_{{g_{n,1} }}^{c} (z,t)} \right) \\ & \quad + \frac{{n_{{e_{n,1} }}^{c} (z,t)}}{{\tau_{eg}^{c} }}\left( {1 - f_{{g_{n,1} }}^{c} (z,t)} \right) - \frac{{n_{{g_{n,1} }}^{c} (z,t)}}{{\tau_{ge}^{c} }}\left( {1 - f_{{e_{n,1} }}^{c} (z,t)} \right) - \frac{{n_{{g_{n,1} }}^{c} (z,t)}}{{\tau_{gr} }} \left( {1 - f_{{g_{n,1} }}^{v} (z,t)} \right) \\ & \quad + \sum\limits_{m = 1}^{2M + 1} {\Gamma L\alpha_{m,n,1}^{1 - band} \frac{{P_{m,1}^{pump} (z,t)}}{{E_{m,1}^{pump} }} \left( {f_{{g_{m,1} }}^{v} (z,t) - f_{{g_{m,1} }}^{c} (z,t)} \right)} \\ & \quad - \sum\limits_{m = 1}^{2M + 1} {\Gamma L\alpha_{m,n,1}^{1 - subband} \frac{{P_{m,1}^{probe} (z,t)}}{{E_{m,1}^{probe} }}\left( {f_{{g_{m,1} }}^{c} - f_{{e_{m,1} }}^{c} } \right)} \\ \end{aligned}$$

8 $$\begin{aligned} \frac{{dn_{{g_{n,2} }}^{c} (z,t)}}{dt} & = \frac{{n_{{g_{n,1} }}^{c} (z,t)}}{{\tau_{down\_trans\_g} }}\left( {1 - f_{{g_{n.2} }}^{c} (z,t)} \right) - \frac{{n_{{g_{n,2} }}^{c} (z,t)}}{{\tau_{up\_trans\_g} }}\left( {1 - f_{{g_{n,1} }}^{c} (z,t)} \right) \\ & \quad + \frac{{n_{{e_{n,2} }}^{c} (z,t)}}{{\tau_{eg}^{c} }}\left( {1 - f_{{g_{n.2} }}^{c} (z,t)} \right) - \frac{{n_{{g_{n,2} }}^{c} (z,t)}}{{\tau_{ge}^{c} }}\left( {1 - f_{{e_{n.2} }}^{c} (z,t)} \right) - \frac{{n_{{g_{n.2} }}^{c} (z,t)}}{{\tau_{gr} }} \left( {1 - f_{{g_{n.2} }}^{v} (z,t)} \right) \\ & \quad + \sum\limits_{m = 1}^{2M + 1} {\Gamma L\alpha_{m,n,2}^{2 - band} \frac{{P_{m,2}^{pump} (z,t)}}{{E_{m,2}^{pump} }}\left( {f_{{g_{m,2} }}^{v} (z,t) - f_{{g_{m,2} }}^{c} (z,t)} \right) } \\ & \quad - \sum\limits_{m = 1}^{2M + 1} {\Gamma L\alpha_{m,n,2}^{2 - subband} \frac{{P_{m,2}^{probe} (z,t)}}{{E_{m,2}^{probe} }}\left( {f_{{g_{m,2} }}^{c} - f_{{e_{m,2} }}^{c} } \right)} \\ \end{aligned}$$

9 $$\begin{aligned} \frac{{dn_{{e_{n,1} }}^{c} (z,t)}}{dt} & = - \frac{{n_{{e_{n,1} }}^{c} (z,t)}}{{\tau_{down\_trans\_g} }}\left( {1 - f_{{e_{n.2} }}^{c} (z,t)} \right) + \frac{{n_{{e_{n,2} }}^{c} (z,t)}}{{\tau_{up\_trans\_g} }}\left( {1 - f_{{e_{n,1} }}^{c} (z,t)} \right)\frac{{n_{{g_{n,1} }}^{c} (z,t)}}{{\tau_{ge}^{c} }}\left( {1 - f_{{e_{n,1} }}^{c} (z,t)} \right) \\ & \quad - \frac{{n_{{e_{n,1} }}^{c} (z,t)}}{{\tau_{eg}^{c} }} \left( {1 - f_{{g_{n,1} }}^{c} (z,t)} \right) - \frac{{n_{{e_{n,1} }}^{c} (z,t)}}{{\tau_{er} }}\left( {1 - f_{{e_{n,1} }}^{v} (z,t)} \right) \\ & \quad + \sum\limits_{m = 1}^{2M + 1} {\Gamma L\alpha_{m,n,1}^{1 - subband} \frac{{P_{m,1}^{probe} (z,t)}}{{E_{m,1}^{probe} }}\left( {f_{{g_{m,1} }}^{c} (z,t) - f_{{e_{m,1} }}^{c} (z,t)} \right)} \\ \end{aligned}$$

10 $$\begin{aligned} \frac{{dn_{{e_{n,2} }}^{c} (z,t)}}{dt} = & & & & \frac{{n_{{e_{n,1} }}^{c} (z,t)}}{{\tau_{down\_trans\_g} }}\left( {1 - f_{{e_{n.2} }}^{c} (z,t)} \right) - \frac{{n_{{e_{n,2} }}^{c} (z,t)}}{{\tau_{up\_trans\_g} }}\left( {1 - f_{{e_{n,1} }}^{c} (z,t)} \right)\frac{{n_{{g_{n,2} }}^{c} (z,t)}}{{\tau_{ge}^{c} }}\left( {1 - f_{{e_{n,2} }}^{c} (z,t)} \right) \\ & - \frac{{n_{{e_{n,2} }}^{c} (z,t)}}{{\tau_{eg}^{c} }} \left( {1 - f_{{g_{n,2} }}^{c} (z,t)} \right) - \frac{{n_{{e_{n,2} }}^{c} (z,t)}}{{\tau_{er} }}\left( {1 - f_{{e_{n,2} }}^{v} (z,t)} \right) \\ & + \sum\limits_{m = 1}^{2M + 1} {\Gamma L\alpha_{m,n,2}^{2 - subband} \frac{{P_{m,2}^{probe} (z,t)}}{{E_{m,2}^{probe} }}\left( {f_{{g_{m,2} }}^{c} (z,t) - f_{{e_{m,2} }}^{c} (z,t)} \right)} \\ \end{aligned}$$

11 $$\begin{aligned} \frac{{dn_{{g_{n,i} }}^{v} (z,t)}}{dt} = & \frac{{n_{{g_{n.i} }}^{c} (z,t)}}{{\tau_{gr} }}\left( {1 - f_{{g_{n.i} }}^{v} } \right) + \frac{{n_{{e_{n,i} }}^{v} (z,t)}}{{\tau_{eg}^{v} }}\left( {1 - f_{{g_{n.i} }}^{v} (z,t)} \right) - \frac{{n_{{g_{n,i} }}^{v} (z,t)}}{{\tau_{ge}^{v} }}\left( {1 - f_{{e_{n.i} }}^{v} (z,t)} \right) \\ & - \sum\limits_{m = 1}^{2M + 1} {\Gamma L\alpha_{m,n,i}^{i - band} \frac{{P_{m,i}^{pump} (z,t)}}{{E_{m,i}^{pump} }} \left( {f_{{g_{m,i} }}^{v} (z,t) - f_{{g_{m,i} }}^{c} (z,t)} \right)} \\ \end{aligned}$$

12 $$\frac{{dn_{{e_{n,i} }}^{v} (z,t)}}{dt} = \frac{{n_{{e_{n.i} }}^{c} (z,t)}}{{\tau_{er} }}\left( {1 - f_{{e_{n.i} }}^{v} (z,t)} \right) + \frac{{n_{{g_{n,i} }}^{v} (z,t)}}{{\tau_{ge}^{v} }} \left( {1 - f_{{e_{n.i} }}^{v} (z,t)} \right) - \frac{{n_{{e_{n,i} }}^{v} (z,t)}}{{\tau_{eg}^{v} }}\left( {1 - f_{{g_{n.i} }}^{v} (z,t)} \right)$$

13 $$\frac{{\partial P_{m,i}^{pump} (z,t)}}{\partial z} = \left( { - \sum\limits_{n = 1}^{2M + 1} {\Gamma \alpha_{m,n,i}^{i - band} \left( {f_{{g_{n,i} }}^{v} (z,t) - f_{{g_{n,i} }}^{c} (z,t)} \right)} - \alpha_{{\text{int}}} } \right)P_{m,i}^{pump} (z,t)$$

14 $$\frac{{\partial P_{m,i}^{probe} (z,t)}}{\partial z} = \left( { - \sum\limits_{n = 1}^{2M + 1} {\Gamma \alpha_{m,n,i}^{i - subband} \left( {f_{{g_{n,i} }}^{c} (z,t) - f_{{e_{n,i} }}^{c} (z,t)} \right)} - \alpha_{{\text{int}}} } \right)P_{m,i}^{probe} (z,t)$$

where $n_{{g_{n,i} }}^{c}$, $n_{{e_{n,i} }}^{c}$, $n_{{g_{n,i} }}^{v}$, and $n_{{e_{n,i} }}^{v}$ are the number of electrons in GS^c^, ES^c^, GS^v^, and ES^v^, respectively. Additionally, $P_{m,i}^{pump}$ and $P_{m,i}^{probe}$ are the optical power of the pump signal and the CW probe signal, respectively. The corresponding carrier occupation probabilities are demonstrated as $f_{{g_{n.i} }}^{c}$, $f_{{e_{n.i} }}^{c}$, $f_{{g_{n.i} }}^{v}$ , and $f_{{e_{n.i} }}^{v}$, respectively. Also, the number of electrons related to their corresponding occupation probabilities as $n_{{g_{n,i} }}^{c(v)} (z,t) = f_{{g_{n,i} }}^{c(v)} (z,t)N_{{G_{i} }}^{c(v)}$, $n_{{e_{n,i} }}^{c(v)} (z,t) = f_{{e_{n,i} }}^{c(v)} (z,t)N_{{E_{i} }}^{c(v)}$. All the time constants are introduced in section ‘The proposed two-channel AOM’, in detail. Besides, the time constants of the electron escape process in the conduction and the valence bands are obtained as

15 $$\tau_{ge}^{c(v)} = \tau_{eg}^{c(v)} \frac{{D_{g}^{c(v)} }}{{D_{e}^{c(v)} }}\exp \left( {\Delta E_{eg}^{c(v)} /KT} \right)$$

where $\Delta E_{{_{eg} }}^{c(v)}$ is the energy spacing between ES^c(v)^ and GS^c(v)^^47–49^. In addition, $D_{g}^{c(v)}$ and $D_{e}^{c(v)}$ are the electron degeneracy of the ground and excited states in the conduction and the valance band, respectively which is determined by $D_{g}^{c} = 2$, $D_{e}^{c} = 6$, $D_{g}^{v} = 2$, and $D_{e}^{v} = 6$^50^. The total number of the electrons of each state, GS^c^, ES^c^, GS^v^, and ES^v^ are demonstrated with $N_{{G_{i} }}^{c(v)} = N_{QD,i} V_{d} D_{g}^{c(v)}$, $N_{{E_{i} }}^{c(v)} = N_{QD,i} V_{d} D_{e}^{c(v)}$, where *V*_*d*_ is the volume of the active region and N_QD,i_ is the corresponding QD density for channel-1 and 2, respectively.

## The performance of two-channel AOM

The two-channel AOM has been characterized by solving the improved rate and propagation Eqs. (7–14). Hence, the active region of the structure is divided into 100 parts in the z-direction and each part is completely analyzed in the time domain. The required parameters and time constants are specified in Table 2. The dynamics of carrier densities as well as the optical power of the pump and probe signals for each region is then calculated. This process continues until the end of the last region. Furthermore, the total interband and intraband absorption coefficient can be obtained as,

16 $$\alpha_{m,i}^{i - band - T} (t) = \int\limits_{0}^{L} {\left[ {\sum\limits_{n = 1}^{2M + 1} {\alpha_{m,n,i}^{i - band} \left( {f_{{g_{n,i} }}^{v} (z,t) - f_{{g_{n,i} }}^{c} (z,t)} \right)} } \right]dz}$$

17 $$\alpha_{m,i}^{i - subband - T} (t) = \int\limits_{0}^{L} {\left[ {\sum\limits_{n = 1}^{2M + 1} {\alpha_{m,n,i}^{i - subband} \left( {f_{{g_{n,i} }}^{c} (z,t) - f_{{e_{n,i} }}^{c} (z,t)} \right)} } \right]dz}$$

**Table 2:** The Parameters used in solving rate and propagation Equation^30,33,47–50^.

Parameters	Channel-1	Channel-2
Radius of QD [nm]	R_1_ = 2	R_2_ = 2.8
L [µm]	150	
W, th [µm]	200	
Volume of QD active region (V_d_) [cm^-3^]	3.375 × 10^–6^	
QD volume density (N_QD_) [cm^-3^]	0.5 × 10^9^	1.5 × 10^9^
The optical confinement factor (Γ)	0.03	
The waveguide intrinsic loss (α_int_) [m^-1^]	2000	
The energy spacing between ES^c^ and GS^c^ (ΔE_eg_^c^) [eV]	0.398	0.246
The energy spacing between ES^v^ and GS^v^ (ΔE_eg_^v^) [eV]	0.144	0.102
The electron relaxation time from the ES^c^ to the GS^c^ (τ_eg_^c^) [ps]	1	
The electron relaxation time from the GS^v^ to the ES^v^ (τ_ge_^v^) [ps]	0.13	
The electron recombination time from the ES^c^ to the ES^v^ (τ_er_) [ps]	1	
The electron recombination time from the GS^c^ to the GS^v^ (τ_gr_) [ps]	1	

The CW input probe power for both channel-1 and channel-2 is equal to 3 mW, both of which are modulated by two pump signals in which their input amplitude is 120 mW and 200 mW for channel-1 and channel-2, respectively. An input Gaussian pulse train with a pulse width of 100 fs is applied at the frequency of 50 GHz. With the first pulse of the pump applied to the modulator, the number of electrons in GS^v^ level decreases. In contrast, the number of electrons at GS^c^ level enhances due to interband absorption. The wavelength of 3 μm as a probe signal is applied simultaneously to the channel-1, and meanwhile, the wavelength of 5 μm probe signal is applied to the channel-2, all of which are carried out in the intersubband absorption from the GS^c^ level to the ES^c^.

**Figure 3:**
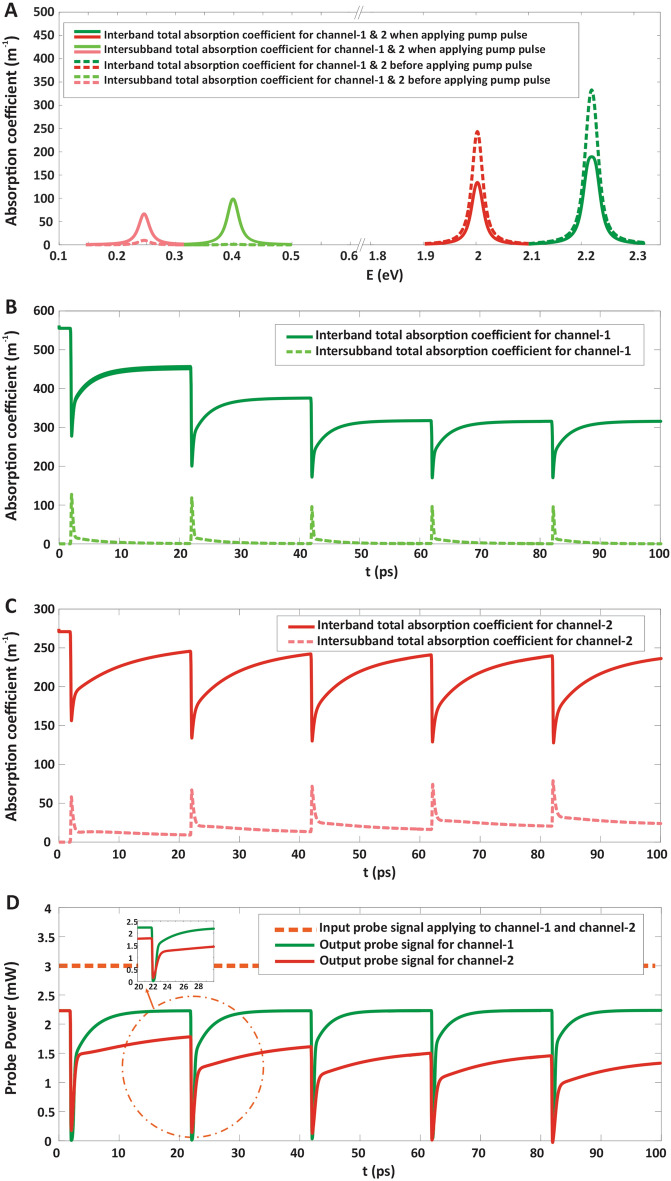
The performance of two-channel AOM. (**A**) The total interband and intersubban absorption coefficient spectrum for channel-1 and channel-2 before and after applying pump pulse, (**B**) and (**C**) Dynamics of the total interband and intersubband absorption coefficient for channel-1 and channel-2, respectively, (**D**) The output modulated probe power in which the input CW probe power is 3 mW for channel-1 and channel-2, by applying pump pulse train at frequency of 50 GHz.

The total interband and intersubband absorption spectrum are obtained using Eqs. (16,17), and have been demonstrated in Fig. 3A at t = 11 ps for channel-1 and channel-2 before applying the pump pulse at t = 12 ps, in which indicated by dashed dark green and dark red lines (interband spectrum) and dashed light green and light red lines (intersubband spectrum) for channel-1 and channel-2, respectively. It is obviously shown that the total intersubband absorption is negligible for both channels due to the lack of carriers in the GS^c^ as a result of pump signal absence. Additionally, the total interband and intersubband absorption spectrum at t = 12 ps (when applying the Gaussian pump with frequency 50 GHz) has been depicted in Fig. 3A by solid dark green and dark red lines (interband spectrum) and solid light green and light red lines (intersubband spectrum) for channel-1 and channel-2, respectively. Applying the pump signal, leads to increasing the total intersubband absorption coefficient. Furthermore, the dynamics of total interband and intersubband absorption for channel-1 and channel-2 have been illustrated in Fig. 3B,C, respectively. Finally, the output modulated probe power is depicted after applying the pump pulse train at the frequency of 50 GHz in Fig. 3D, in which the input CW probe power is 3mW for both channel-1 and channel-2.

One of the most essential factors in the characteristics of a modulator is the depth of modulation, which is defined as the change in the amplitude of the probe signals when modulation is operated. When the pump signal is applied to the modulator, the applied probe signal simultaneously starts to absorb and just then the probe power is absorbed, the output signal turns to “OFF” state. When the pump is turned off, because the interband absorption process is slowing down, the probe’s power will not be completely absorbed, and the output probe will be turned “ON”. So, the amplitude of the probe power (carrier wave) changes when the information signal (pump power) changes, and the highest change in the amplitude is specified as the MD in the modulation process. The MD is calculated by the difference between “ON-state” output power and “OFF-state” output power which is obtained as^51,52^,

18 $$MD = \frac{{P_{ON} - P_{OFF} }}{{P_{ON} }}$$

**Figure 4:**
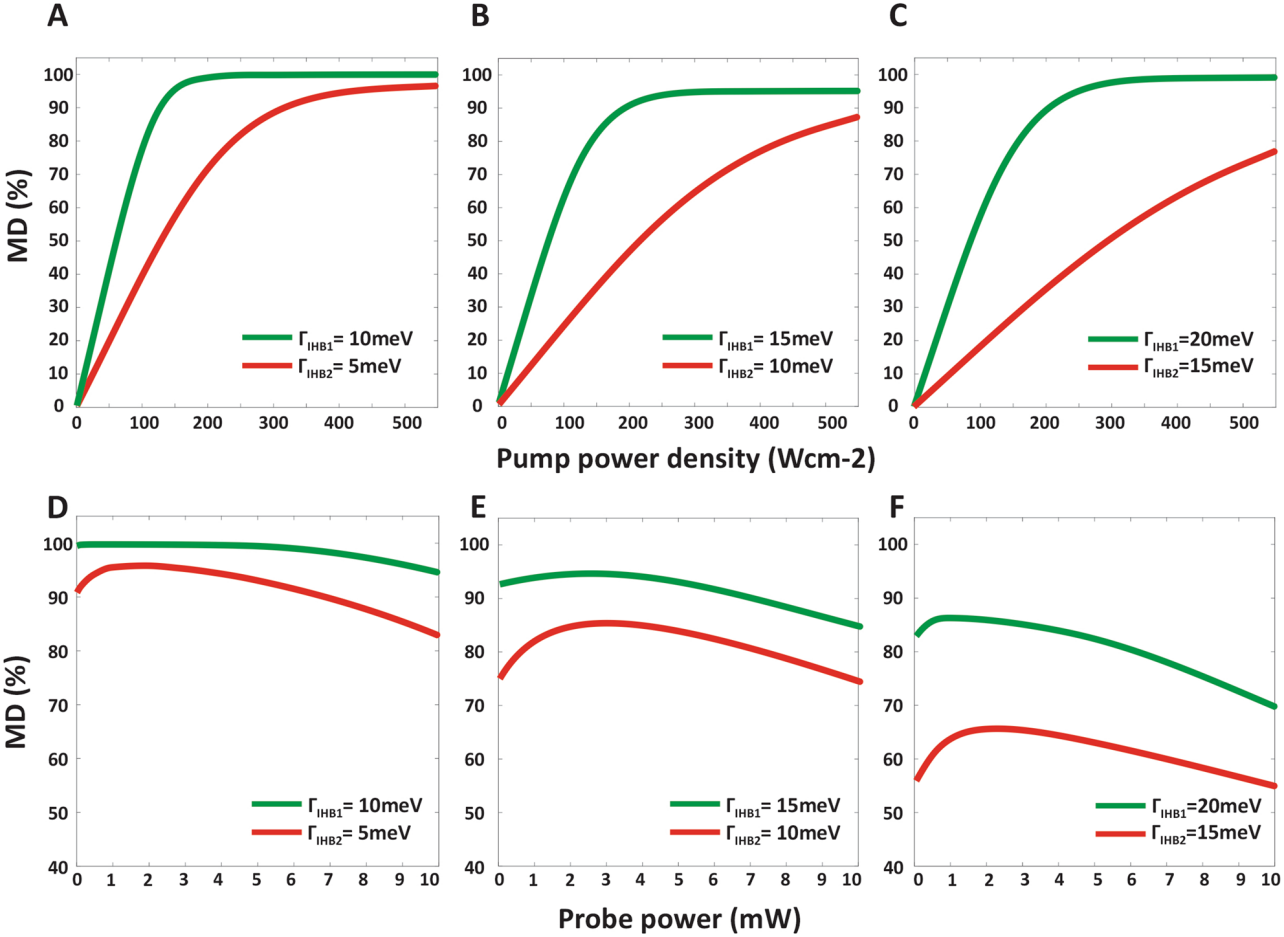
The dependance of MD on pump and probe power. (**A**) The MD as a function of input pump power density at the frequency of 50 GHz and the probe power of 3 mW for both channel-1 and channel-2. The FWHM of IHB is assumed to Γ_IHB1_ = 10 meV, Γ_IHB2_ = 5 meV, (**B**) Γ_IHB1_ = 15 meV, Γ_IHB2_ = 10 meV, and (**C**) Γ_IHB1_ = 20 meV, Γ_IHB2_ = 15 meV for channel-1 and channel-2, respectively. (**D**) The MD as a function of input probe power values at the fix pump power densities of 300 Wcm^−2^ and 500 Wcm^−2^ for channel-1 and channel-2, respectively and frequency of 50 GHz for both channel-1 and channel-2. The FWHM of IHB is assumed to Γ_IHB1_ = 10 meV, Γ_IHB2_ = 5 meV, (**E**) Γ_IHB1_ = 15 meV, Γ_IHB2_ = 10 meV, and (**F**) Γ_IHB1_ = 20 meV, Γ_IHB2_ = 15 meV for channel-1 and channel-2, respectively.

The MD versus the pump power density at the frequency of 50 GHz, and the probe power of 3 mW for both channel-1 (solid green line) and channel-2 (solid red line), have been shown for different values of FWHM of the IHB, in Fig. 4A–C. It is observed that by enhancing the pump power, the MD increased as a result of growing the number of carriers at the GS^c^. However, the MD will be saturated when the pump power gets larger values. In other words, because the GS^c^ level is completely filled, MD will not be affected by the pump power after this increase. It is also observed that with increasing $\Gamma_{IHB}$ (IHB effect), the MD value decreases. However, increasing $\Gamma_{IHB}$ which is equivalent to reducing the accuracy of the fabricating process through the solution-process method, the performance of both channels simultaneously is acceptable, and the MD above 60% can be achieved. The greater the radial distribution relative to the central radius of the QDs, the lower the MD based on the obtained results shown in Fig. 4A–C. Finally, according to Fig. 4B, for optimal modulation, pump power densities are set at 300 Wcm^−2^ and 500 Wcm^−2^ and $\Gamma_{IHB}$ is considered 15 meV and 10 meV for channel-1 and channel-2, respectively.

Figure 4D–F shows MD as a function of different input probe power at the frequency of 50 GHz and at the pump power density of 300 Wcm^−2^ and 500 Wcm^−2^ for channels-1 and channel-2, respectively. This figure shows that by increasing the power of the input probe, the MD can be increased. However, as shown in the figure, with further increase of the probe power from the threshold power, the MD decreases. This is because with increasing the probe power, more carriers are absorbed in the intersubband, and until the ES^c^ reaches its saturation value (is not fully filled), this procedure increases MD. But after the saturation of the ES^c^, the process of intersubband absorption is reduced, so by increasing the probe power, not only does not the absorption rise, but on the contrary, the probe signal is transmitted to the output without absorption, and this reduces the MD. As the Fig. 4D–F shows, the MD is plotted according to the probe power for different $\Gamma_{IHB}$ s, indicating that the MD decreases with increasing $\Gamma_{IHB}$. Finally, according to Fig. 4E for optimal modulation, probe power is set to be 3 mW for both channels and $\Gamma_{IHB}$ is considered 15 meV and 10 meV for channel-1 and channel-2, respectively.

Eventually, should be paid attention to this fact that decreasing the accuracy of fabricating procedures leads to reducing the manufacturing costs. So, for a trade-off between high MD and low-cost manufacturing, the values of 3 mW for both channels probe power, 300 Wcm^−2^ and 500 Wcm^−2^ for pump power densities with 15 meV and 10 meV for $\Gamma_{IHB}$ are opted for channel-1 and channel-2, respectively.

**Figure 5:**
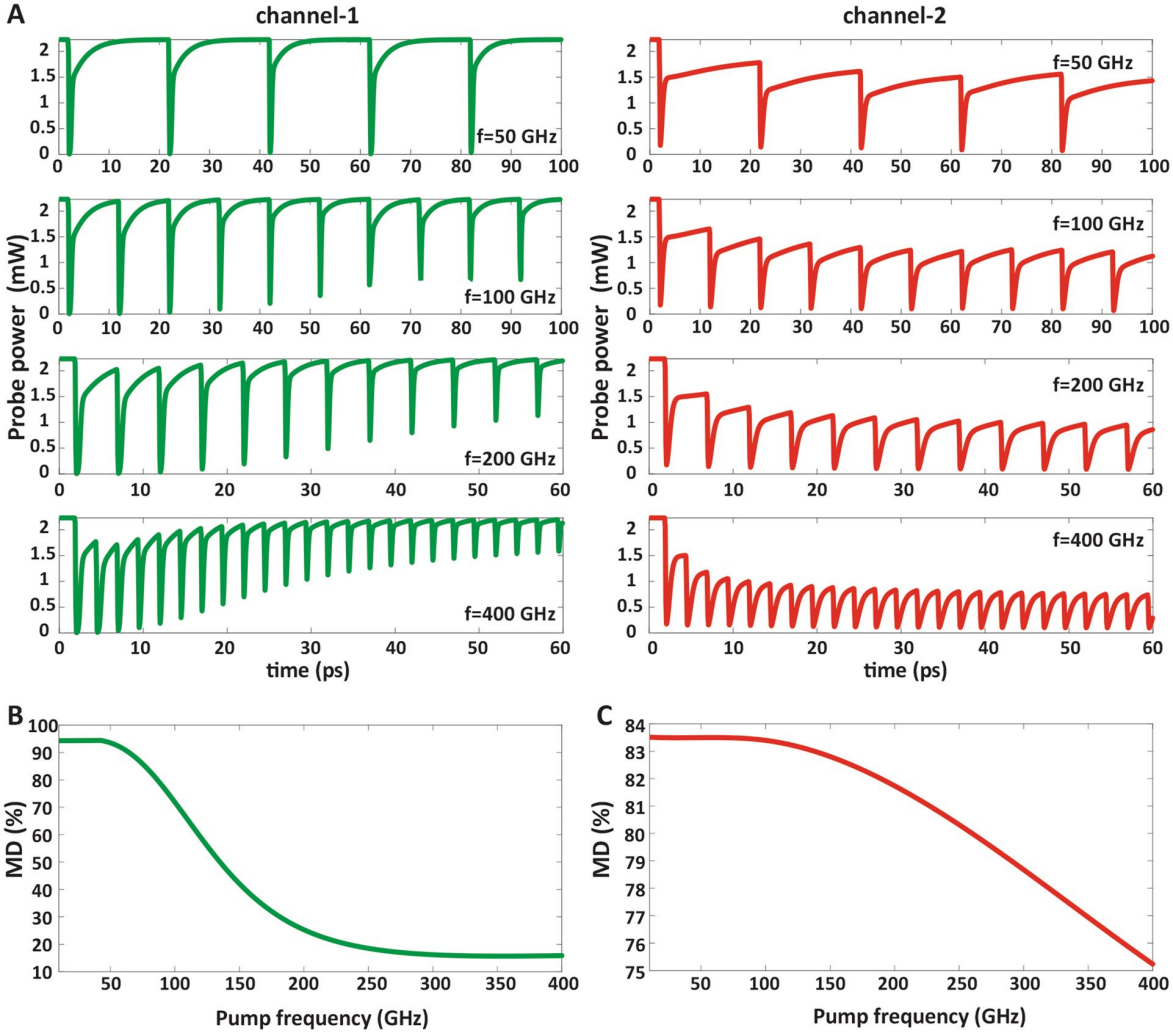
The dependance of MD on the frequency of applied pump power. (**A**) The output modulated probe power at different frequencies, which is indicated that by increasing the modulation frequency, MD and the dynamic range between P_ON_ and P_OFF_ in the probe power signal decreases. (**B**) The MD as a function of pump power frequency at the pump power densities of 300 Wcm^−2^ and 500 Wcm^−2^ for channel-1 and (**C**) for channel-2, respectively and probe power of 3 mW for both channel-1 and channel-2.

In Fig. 5A–C, the output modulated probe power and MD at different pump pulse frequencies are plotted for channel-1 in the left column and for channel-2 in the right column. It is indicated that by increasing the modulation frequency, MD and the dynamic range between P_ON_ and P_OFF_ in the probe power signal decreases.

As shown in Fig. 5A, in channel-1 carriers of conductive band descend due to the FRET process so the probe power cannot be absorbed completely and the output probe power do not turn to “OFF-state” perfectly. Therefore, the output probe signal will have a larger amplitude than its minimum value in “OFF-state”. Conversely in channel-2, carriers of conductive band ascend due to the FRET process so the probe power cannot pass completely and the output probe power do not turn to “ON-state” perfectly because the system does not have enough time to reach its full recovery time. As a result, the output probe signal will have a smaller amplitude than its maximum value in “ON-state”.

It should be noted that by increasing the input pump power densities nonlinear phenomenon, two-photon-absorption, occurs preventing the probe absorption by the pump. Therefore, QDs become transparent to either the incoming pump or probe signal and no more probe power is absorbed. As a result, the modulator performance which is practically based on interband and intraband absorption is totally disrupted. The maximum power density applied to the proposed device is limited to 600 Wcm^−2^ ^13^.

## Conclusion

In this paper, a novel model to design a two-channel AOM based on solution-processed CdSe/ZnS QD structure has been introduced. Due to the quantum size effect in the quantum-based devices, tunable absorption spectrum can be achieved, so this AOM is modeled for two sizes of QDs to modulate two wavelengths of MIR spectra (3 µm and 5 µm). The proposed two-channel AOM has been modeled in the coupled rate and propagation equations framework, considering homogeneous and inhomogeneous broadenings. It is shown that the MD for the proposed AOM can be obtained approximately at 94% and 83.5% for channel-1 and channel-2, respectively, when the 300 Wcm^−2^ and 500 Wcm^−2^ pump power density at the 50 GHz frequency are applied to channel-1 and channel-2 and the input probe power is 3 mW for both channels. The simulation results demonstrate that the MD decreases by increasing the IHB so, it is essential to reduce the IHB effect as much as possible by synthesis of QDs with higher accuracy and resolution.

## Data Availability

The datasets used and/or analyzed during the current study are available from the corresponding author on reasonable request.
